# Supplementary material for: Group additivity-Pourbaix diagrams advocate thermodynamically stable nanoscale clusters in aqueous environments
Source: Nat Commun. 2017 Jun 15;8:15852. doi: 10.1038/ncomms15852 (PMC5481758; doi:10.1038/ncomms15852)
Supplement: Supplementary Information [file ncomms15852-s1.pdf]

Type of file: PDF
Size of file: 0 KB
Title of file for HTML: Supplementary Information
Description: Supplementary Figures, Supplementary Tables, Supplementary Notes, Supplementary Method and Supplementary References

Type of file: PDF
Size of file: 0 KB
Title of file for HTML: Peer Review File
Description:

Supplementary Figure 1. GA predicted versus QM computed hydrolysis energies.

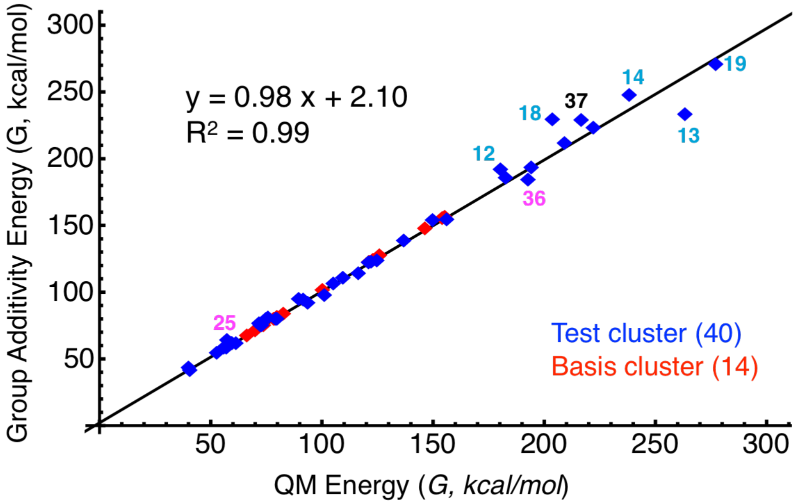

The slope of the linear regression line is near unity (0.98), the intercept is 2.10 and the R^2 value is 0.99. Clusters whose error are larger than 5 kcal/mol are labeled. Al clusters are in cyan, Ga in magenta, and heterometallic clusters in black.

Supplementary Figure 2. The structures of the 36 test clusters.

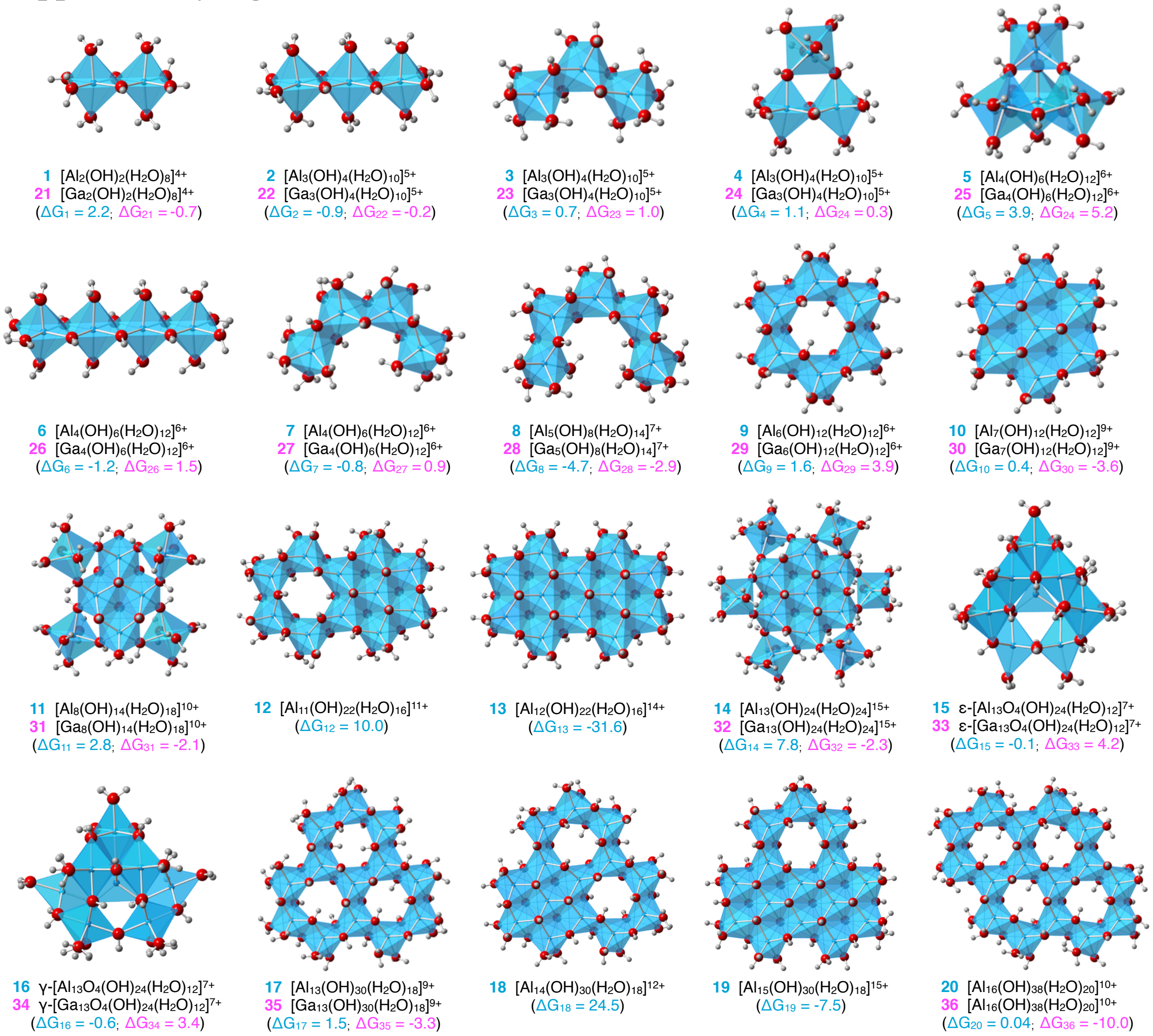

$\Delta G_{\text{total,GA-QM}}$ is the difference between the GA predicted and QM computed Gibbs free energies. Energies shown here are obtained by QM level of theory I and are in the unit of kcal·mol^{-1}. The notation for Al clusters are shown in cyan, magenta notation represents Ga clusters, and the black for heterometallic clusters.

Supplementary Table 1. The Gibbs free energies of metal cation (M^{3+}) and μ_4-O, μ_3-OH, μ_2-OH$_{edge,O}$, μ_2-OH$_{edge,OH}$, μ_2-OH$_{edge,core\text{-}links}$, μ_2-OH$_{corner}$, and η-H_2O in Al and Ga aquo-oxo/hydroxo clusters.

	$[Al_mO_x(OH)_y(H_2O)_z]^{(3m\text{-}2x\text{-}y)}$ (a.u.)	$Ga_mO_x(OH)_y(H_2O)_z]^{(3m\text{-}2x\text{-}y)}$ (a.u.)
M^{3+}	-241.278336	-1923.603085
μ_4-O	-76.143619	-76.166418
μ_3-OH	-76.450168	-76.442767
μ_2-OH$_{edge,O}$	-76.359781	-76.340185
μ_2-OH$_{edge,OH}$	-76.313202	-76.305428
μ_2-OH$_{edge,core\text{-}links}$	-76.315207	-76.308287
μ_2-OH$_{corner}$	-76.313110	-76.308385
η-H_2O	-76.587968	-76.584082

Supplementary Table 2. The species and their quantities contained in each aluminum hydroxyl cluster studied in this manuscript.

	μ_4-O	μ_3-OH	μ_2-OH (edge,O^{2-})	μ_2-OH (edge,OH^-)	μ_2-OH (edge, core-links)	μ_2-OH (corner)	η-H_2O	Ref.
a_1, a_2		2		4			10	[1,2]
b_1, b_2				2		4	12	[3–5]
c_1, c_2				6			12	[6]
d_1, d_2		6		6		2	14	
e_1, e_2	4		11	2		10	14	
f_1, f_2	4		12	6		6	12	[7–9]
g_1, g_2					22		16	[10,11]
1, 21				2			8	[12]
2, 22				4			10	[13]
3, 23				4			10	[10]
4, 24				2		2	10	[3,4,13]
5, 25						6	12	[14]
6, 26				6			12	
7, 27				6			12	
8, 28				8			14	
9, 29					12		12	[10,11,15]
10, 30		6		6			12	[16–19]

11, 31		2		4		8	18	20
12		6		6	10		16	21,22
13		12		10			16	22,23
14, 32		6		6		12	24	22,24–27
15, 33	4		12	12			12	9,28,29
16, 34	4		12	2		10	12	9,30
17, 35					30		18	31
18		6		6	18		18	21,22
19		12		10	8		18	21,22
20, 36					38		20	10,11
37		6		6		12	24	32
38		6		6		12	24	32
39	4		12	12			12	33,34
40	4		12	12			12	

Supplementary Table 3. The QM computed and GA predicted equilibrium constants of $[Al(OH)_4]^-$, $[Ga(OH)_4]^-$, Al_{13}^k and Ga_{13}^k clusters as well as H_2O disproportionation.

	Exp.	QM	GA
Al_{13}^k	98.73[35]		
ε-isomer		99.32	99.28
δ-isomer		99.53	99.53
γ-isomer		100.15	99.70
Ga_{13}^k	62.34[36]		
γ-isomer		63.11	65.63
ε-isomer		76.13	79.23
δ-isomer		71.07	71.07
$[Al(OH)_4]^-$	23.0[37]	25.06	—
$[Ga(OH)_4]^-$	16.6[37]	16.72	—
H_2O	14.0[35]	14.49	—

Supplementary Table 4. The chemical potential ($\mu_{\text{cluster}}^{\text{ref}}$) of all the clusters at the standard state (room temperature 25 °C, atmospheric pressure, and 1 M concentration).

	$\mu_{\text{cluster}}^{\text{ref}}$ kcal·mol^{-1}		$\mu_{\text{cluster}}^{\text{ref}}$ kcal·mol^{-1}
1	-753.33	20	-4896.56
2	-1076.41	21	-605.55
3	-1076.41	22	-854.79
4	-1076.31	23	-854.79
5	-1399.18	24	-858.49
6	-1399.48	25	-1115.22
7	-1399.48	26	-1104.02
8	-1722.66	27	-1104.02
9	-1953.85	28	-1353.36
10	-2024.27	29	-1517.01
11	-2576.70	30	-1523.28
12	-3222.29	31	-2006.38
13	-3295.30	32	-3040.99
14	-3962.32	33	-2685.67
15 (Al_{13}^{7+})	-3633.79	34 (Ga_{13}^{7+})	-2704.27
16	-3633.29	35	-3079.99
17	-4024.22	36	-3735.02
18	-4094.63	37 (Al_{26}^{12+})	-7155.00
19	-4167.64		

Supplementary Note 1

$$G_{\mathrm{a}} - 4G_{M^{3+}} = 2G_{\mu_3\text{-OH}} + 4G_{\mu_2\text{-OH}_{\mathrm{edge,OH}}} + 10G_{\eta\text{-H}_2\mathrm{O}} \quad (1)$$

$$G_{\mathrm{b}} - 4G_{M^{3+}} = 2G_{\mu_2\text{-OH}_{\mathrm{edge,OH}}} + 4G_{\mu_2\text{-OH}_{\mathrm{edge,OH}}} + 12G_{\eta\text{-H}_2\mathrm{O}} \quad (2)$$

$$G_{\mathrm{c}} - 4G_{M^{3+}} = 6G_{\mu_2\text{-OH}_{\mathrm{edge,OH}}} + 12G_{\eta\text{-H}_2\mathrm{O}} \quad (3)$$

$$G_{\mathrm{d}} - 8G_{M^{3+}} = 6G_{\mu_3\text{-OH}} + 6G_{\mu_2\text{-OH}_{\mathrm{edge,OH}}} + 2G_{\mu_2\text{-OH}_{\mathrm{corner}}} + 14G_{\eta\text{-H}_2\mathrm{O}} \quad (4)$$

$$G_{\mathrm{e}} - 13G_{M^{3+}} = 4G_{\mu_4\text{-O}} + 11G_{\mu_2\text{-OH}_{\mathrm{edge,O}}} + 2G_{\mu_2\text{-OH}_{\mathrm{edge,OH}}} + 10G_{\mu_2\text{-OH}_{\mathrm{corner}}} + 14G_{\eta\mathrm{H}_2\mathrm{O}} \quad (5)$$

$$G_{\mathrm{f}} - 13G_{M^{3+}} = 4G_{\mu_4\text{-O}} + 12G_{\mu_2\text{-OH}_{\mathrm{edge,O}}} + 6G_{\mu_2\text{-OH}_{\mathrm{edge,OH}}} + 6G_{\mu_2\text{-OH}_{\mathrm{corner}}} + 12G_{\eta\mathrm{H}_2\mathrm{O}} \quad (6)$$

$$G_{\mathrm{g}} - 10G_{M^{3+}} = 22G_{\mu_2\text{-OH}_{\mathrm{edge,core\text{-}links}}} + 16G_{\eta\text{-H}_2\mathrm{O}} \quad (7)$$

$$G_{\mathrm{ligand},M_xN_y} = \alpha G_{\mathrm{ligand},M_{(x+y)}} + \beta G_{\mathrm{ligand},n_{(x+y)}} \quad (8)$$

Supplementary Method

The ground-state structures and Gibbs free energy are computed by HF/6-31G(d,p) with IEFPCM-UFF continuum solvation model for water. The electronic energy was refined using B3LYP/6-311+G(d) single point, and the solvation energy was recomputed using HF/6-311+G(d) with CPCM-UAKS for water. As an example, Supplementary Equation 1-7 show the method to calculate the energy of structure a-f.

These seven basis ligands are η–H_2O (the aqua ligands that fill the peripheral coordination sites); μ_4-O; μ_3-OH; μ_2-OH_{corner} (binds two metallic cations which share one common hydroxo ligand at the corner sites of the metallic octahedral structure), μ_2-$OH_{edge,O}$ (binds two metallic cations which share one common hydroxo ligand and one common oxo ligand at the same edge of the metallic octahedral structure), μ_2-$OH_{edge,OH}$ (binds two metallic cations which share two common hydroxo ligand at the same edge of the metallic octahedral structure), and μ_2-$OH_{edge,\ core\text{-}links}$, (μ_2-$OH_{edge,OH}$ ligands in gibbsite-like core-links structures).

Supplementary Note 2

Accuracy of group addivity method. The regression plot comparing the GA predicted and QM computed hydrolysis energies is shown in Supplementary Figure 1. The linearity between the QM energy and GA predicted energy (R^2 value =0.99) shows that the GA method robustly and accurately reproduces the QM computed hydrolysis reaction energies of Al, Ga and Al/Ga aqueous oxo/hydroxo clusters.

Supplementary Table 3 lists the experimental QM computed and predicted equilibrium constants of selected clusters. The GA method reproduces QM computed and experimental data.

The Gibbs free energies of heterometallic flat and Keggin tridecamers are predicted and the results are shown in Supplementary Figure 2. In computing the GA energy for heterometallic clusters, the ligand energies of homometallic clusters are used for ligands attached to only one type of metal cation. If the ligand bridges two different cations, the

ligand energy is computed from the energies of that species of ligand in both of the homometallic clusters by specific weighting ratio as shown in Supplementary Equation 8.

The weighting ratios differ from species of ligands and are closely related to the cluster structures. For bridging μ_2-OH_{corner} ligands in the heterometallic flat tridecamers (x = 7, y = 6), $\alpha = \beta = 0.5$; for bridging μ_4-O ligand in Keggin tridecamers (x = 1, y = 12), $\alpha = 0$ and $\beta = 1$.

The above assumption gave good predictions for the Gibbs free energy of Ga_7Al_6 ($\Delta G_{total,GA\text{-}QM}$ = 1.3 kcal·mol^{-1}), $GaAl_{12}$ ($\Delta G_{total,GA\text{-}QM}$ = 0.1 kcal·mol^{-1}) and $AlGa_{12}$ ($\Delta G_{total,GA\text{-}QM}$ = 0.0 kcal·mol^{-1}). The error of Al_7Ga_6 is somewhat larger ($\Delta G_{total,GA\text{-}QM}$ = 11.0 kcal·mol^{-1}). The origin of rather larger error in this case is the μ_2-OH_{corner} ligand, which is overestimated by 0.91 kcal·mol^{-1}. These ligands in Al_7Ga_6 are slightly closer to the $[Al_7(OH)_{12}]^{9+}$ core (1.86 Å in average) than the analogous ligands are to the $[Ga_7(OH)_{12}]^{9+}$ core in Ga_7Al_6 (1.92 Å in average). Hence, the μ_2-OH_{corner} ligands in Al_7Ga_6 experience stronger electrostatic attraction from $[Al_7(OH)_{12}]^{9+}$ core and is consequently slightly lower in energy than the μ_2-OH_{corner} ligands of flat Al_{13} and Ga_{13}.

In Keggin tridecamers, the available space that the μ_4-O ligands can occupy is strictly confined by the central tetrahedral cation and the outer octahedral cations. Since the cluster size of Keggin tridecamers only has negligible change with substitution of the cation in the tetrahedral core and both Ga and Al cations are trivalent, it is not surprising that the energy of bridging μ_4-O ligand is determined exclusively by the outer octahedral metallic cations.

The difference between the GA predicted and QM computed Gibbs free energies ($\Delta G_{\mathrm{total,GA\text{-}QM}}$) of 40 clusters are shown in Supplementary Figure 2. The mean absolute error (*MAE*) is 4.0 kcal·mol^{-1} (standard deviation = 6.3 kcal·mol^{-1}). The mean absolute percent error (*MAPE*) for the absolute energies of the cluster is 0.0001% (standard deviation = 0.00017%). Among all the clusters, $[Al_{12}(OH)_{22}(H_2O)_{16}]^{14+}$ (cluster **13** in Supplementary Figure 2), has the largest error, -31.6 kcal·mol^{-1} or 0.00087% of the total energy. The *MAPE* for the hydrolysis energies of the clusters is 2.9% (standard deviation = 2.9 %).

Supplementary References

1. Miyasaka, H., Nakata, K., Sugiura, K., Yamashita, M. & Clérac, R. A Three-Dimensional Ferrimagnet Composed of Mixed-Valence Mn4 Clusters Linked by an{Mn[N(CN)2]6}4− Unit. *Angew. Chemie Int. Ed.* **43,** 707–711 (2004).

2. Yan, P.-F. *et al.* Planar Tetranuclear Dy(III) Single-Molecule Magnet and Its Sm(III), Gd(III), and Tb(III) Analogues Encapsulated by Salen-Type and β-Diketonate Ligands. *Inorg. Chem.* **50,** 7059–7065 (2011).

3. Stuenzi, H., Spiccia, L., Rotzinger, F. P. & Marty, W. Early stages of the hydrolysis of chromium(III) in aqueous solution. 4. The stability constants of the hydrolytic dimer, trimer, and tetramer at 25 C and I = 1.0 M. *Inorg. Chem.* **28,** 66–71 (1989).

4. Andersen, P., Damhus, T., Pedersen, E., Petersen, A. & Ohtaki, H. Synthesis and Structural, Magnetic, and ESR Characterization of the Tri- and Tetranuclear Hydroxo-Bridged Chromium(III) Ammine Complexes [Cr3(NH3)10(OH)4]Br5.3H2O, [Cr{(OH)2Cr(NH3)4}3]Br6.aq and [Cr4(NH3)12(OH)6]Cl6.4H2O (Rhodoso Chloride). *Acta Chem. Scand.* **38a,** 359–

376 (1984).

5. Bang, E., Lindqvist, O., Boyce, J. B., Claeson, T. & Ohtaki, H. Redetermination of the Crystal Structure of the Tetrameric Chromium(III) Ammine Complex [Cr4(NH3)12(OH)6]Cl6.4H2O at 110 K. *Acta Chem. Scand.* **38a,** 419–421 (1984).

6. Bernal, I., Cetrullo, J. & Berhane, S. THE CRYSTAL AND MOLECULAR STRUCTURES OF TWO DERIVATIVES OF WERNER'S HEXOL CLUSTER CATION: [Co{(OH) 2 Co(NH 3) 4 } 3](NO 3) 5 (OH) · 4H 2 O(I) AND [Co{(OH) 2 Co(NH 3) 4 } 3](NO 3) 6 · 2H 2 O(II). *J. Coord. Chem.* **52,** 185–205 (2000).

7. Rowsell, J. & Nazar, L. F. Speciation and thermal transformation in alumina sols: Structures of the polyhydroxyoxoaluminum cluster [Al30O8(OH)56(H2O)26]18+ and its keggin moiete [4]. *J. Am. Chem. Soc.* **122,** 3777–3778 (2000).

8. Abeysinghe, S., Unruh, D. K. & Forbes, T. Z. Crystallization of Keggin-Type Polyaluminum Species by Supramolecular Interactions with Disulfonate Anions. *Cryst. Growth Des.* **12,** 2044–2051 (2012).

9. Bradley, S. M., Kydd, R. A. & Yamdagni, R. Detection of a new polymeric species formed through the hydrolysis of gallium(III) salt solutions. *J. Chem. Soc. Dalt. Trans.* **1989,** 413 (1990).

10. Stol, R. ., Van Helden, A. . & De Bruyn, P. . Hydrolysis-precipitation studies of aluminum (III) solutions. 2. A kinetic study and model. *J. Colloid Interface Sci.* **57,** 115–131 (1976).

11. Bersillon, J., Brown, D., Fiessinger, F. & Hem, J. Iron in water near wastewater lagoons in Yellowstone National Park, Wyoming. *J. Res. U. S. Geol. Surv.* **6,** 325–

337 (1978).

12. Johansson, G. *et al*. The Crystal Structures of [Al2(OH)2(H2O)8](SO4)2.2H2O and [Al2(OH)2(H2O)8](SeO4)2.2H2O. *Acta Chem. Scand.* **16,** 403–420 (1962).

13. Finholt, J. E., Thompson, M. E. & Connick, R. E. Hydrolytic polymerization of chromium(III). 2. A trimeric species. *Inorg. Chem.* **20,** 4151–4155 (1981).

14. Sun, Z., Wang, H., Feng, H., Zhang, Y. & Du, S. Crystal Structure of [Al 4 (OH) 6 (H 2 O) 12][Al(H 2 O) 6] 2 Br 12 : A New Polyaluminum Compound. *Inorg. Chem.* **50,** 9238–9242 (2011).

15. Murugesu, M., Abboud, K. A. & Christou, G. Preparation and properties of new Fe6 and Fe8 clusters of iron(iii) with tripodal ligands. *Dalt. Trans.* 4552 (2003).

16. Abbati, G. L., Cornia, A., Fabretti, A. C., Caneschi, A. & Gatteschi, D. Structure and Magnetic Properties of a Mixed-Valence Heptanuclear Manganese Cluster. *Inorg. Chem.* **37,** 3759–3766 (1998).

17. Bolcar, M. A., Aubin, S. M. J., Folting, K., Hendrickson, D. N. & Christou, G. A new manganese cluster topology capable of yielding high-spin species: mixed-valence [Mn7(OH)3Cl3(hmp)9]2 + with S ≥ 10. *Chem. Commun.* 1485–1486 (1997).

18. Oshio, H. *et al*. High-Spin Wheel of a Heptanuclear Mixed-Valent FeII,III Complex. *Angew. Chemie Int. Ed.* **42,** 223–225 (2003).

19. Tesmer, M., Müller, B. & Vahrenkamp, H. Oligonuclear zinc complexes of 2-pyridylmethanol. *Chem. Commun.* 721–722 (1997).

20. Casey, W. H., Olmstead, M. M. & Phillips, B. L. A New Aluminum Hydroxide

Octamer, [Al 8 (OH) 14 (H 2 O) 18](SO 4) 5 ·16H 2 O. *Inorg. Chem.* **44,** 4888–4890 (2005).

21. Balan, E., Lazzeri, M., Morin, G. & Mauri, F. First-principles study of the OH-stretching modes of gibbsite. *Am. Mineral.* **91,** 115–119 (2006).

22. Goodwin, J. C., Teat, S. J. & Heath, S. L. How Do Clusters Grow? The Synthesis and Structure of Polynuclear Hydroxide Gallium(III) Clusters. *Angew. Chemie Int. Ed.* **43,** 4037–4041 (2004).

23. Duffy, T. S., Meade, C., Yingwei Fei, Ho-Kwang Mao & Hemley, R. J. High-pressure phase transition in brucite, Mg(OH)2. *Am. Mineral.* **80,** 222–230 (1995).

24. Seichter, W., Mögel, H.-J., Brand, P. & Salah, D. Crystal Structure and Formation of the Aluminium Hydroxide Chloride [Al13(OH)24(H2O)24]Cl15 · 13 H2O. *Eur. J. Inorg. Chem.* **1998,** 795–797 (1998).

25. Gatlin, J. T., Mensinger, Z. L., Zakharov, L. N., MacInnes, D. & Johnson, D. W. Facile Synthesis of the Tridecameric Al 13 Nanocluster Al 13 (μ 3 -OH) 6 (μ 2 -OH) 18 (H 2 O) 24 (NO 3) 15. *Inorg. Chem.* **47,** 1267–1269 (2008).

26. Wang, W., Wentz, K. M., Hayes, S. E., Johnson, D. W. & Keszler, D. A. Synthesis of the Hydroxide Cluster [Al13(μ3-OH)6(μ-OH)18(H2O)24]15+ from an Aqueous Solution. *Inorg. Chem.* **50,** 4683–4685 (2011).

27. Rather, E. *et al.* A Simple Organic Reaction Mediates the Crystallization of the Inorganic Nanocluster [Ga 13 (μ 3 -OH) 6 (μ 2 -OH) 18 (H 2 O) 24](NO 3) 15. *J. Am. Chem. Soc.* **127,** 3242–3243 (2005).

28. Johansson, G., Lundgren, G., Sillén, L. G. & Söderquist, R. On the Crystal Structure

of a Basic Aluminium Sulfate and the Corresponding Selenate. *Acta Chem. Scand.* **14,** 769–771 (1960).

29. Johansson, G., Gullman, L.-O., Kjekshus, A. & Söderquist, R. On the Crystal Structure of Some Basic Aluminium Salts. *Acta Chem. Scand.* **14,** 771–773 (1960).

30. Smart, S. E., Vaughn, J., Pappas, I. & Pan, L. Controlled step-wise isomerization of the Keggin-type Al13 and determination of the γ-Al13 structure. *Chem. Commun.* **49,** 11352 (2013).

31. Pascual-Cosp, J., Artiaga, R., Corpas-Iglesias, F. & Benítez-Guerrero, M. Synthesis and characterization of a new aluminium-based compound. *Dalt. Trans.* 6299 (2009). doi:10.1039/b900550a

32. Mensinger, Z. L., Wang, W., Keszler, D. a. & Johnson, D. W. Oligomeric group 13 hydroxide compounds—a rare but varied class of molecules. *Chem. Soc. Rev.* **41,** 1019–1030 (2012).

33. Lee, A. P., Phillips, B. L., Olmstead, M. M. & Casey, W. H. Synthesis and Characterization of the GeO 4 Al 12 (OH) 24 (OH 2) 12 8+ Polyoxocation. *Inorg. Chem.* **40,** 4485–4487 (2001).

34. Son, J. H. & Kwon, Y. U. Single crystal structure of pure inorganic nanocomposite [GaO4Al12(OH)(24)(H2O)(12)][Al(OH)(6)Mo6O18](2)(OH)center dot 30H(2)O. *Bull. Korean Chem. Soc.* **22,** 1224–1230 (2001).

35. Furrer, G., Trusch, B. & Müller, C. The formation of polynuclear Al13 under simulated natural conditions. *Geochim. Cosmochim. Acta* **56,** 3831–3838 (1992).

36. Frisch, M. J. *et al.* Gaussian 09. (2009).

37. Baes, C. F. & Mesmer, R. E. *The Hydrolysis of Cations*. (Krieger Pub Co, 1986).
